# Graph Neural Network-Based Drug Gene Interactions of Wnt/β-Catenin Pathway in Bone Formation

**DOI:** 10.7759/cureus.68669

**Published:** 2024-09-04

**Authors:** Pradeep Kumar Yadalam, R Ramya, Raghavendra Vamsi Anegundi, Shubhangini Chatterjee

**Affiliations:** 1 Department of Periodontics, Saveetha Dental College and Hospital, Saveetha Institute of Medical and Technical Sciences, Saveetha University, Chennai, IND; 2 Department of Oral Biology, Saveetha Dental College and Hospital, Saveetha Institute of Medical and Technical Sciences, Saveetha University, Chennai, IND

**Keywords:** beta-catenin, bone, drug-gene, graph neural networks, wnt

## Abstract

Introduction

The Wnt/β-catenin pathway is crucial for bone formation and remodeling, regulating osteoblast differentiation, bone remodeling, and skeletal homeostasis. Dysregulation of the Wnt/β-catenin pathway is linked to bone-related diseases like osteoporosis, osteoarthritis, and osteosarcoma. The strategies to modulate this pathway include Wnt agonists, inhibitors, and small molecules. Graph neural networks (GNNs) have shown potential in understanding drug-gene interactions, providing accurate predictions, identifying novel drug-target pairs, and enabling personalized drug therapy. So we aim to predict GNN-based drug-gene interactions of Wnt/β-catenin pathway in bone formation.

Methodology

The drug-gene interactions of Wnt signaling were annotated and preprocessed using Cytoscape, a powerful tool for building drug-gene interactions. Data was imported, nodes representing drugs and genes were created, and edges represented their interactions. GNNs were used to prepare data for nodes, genes, and drugs. GNNs are designed to operate on graph-structured data, capable of learning complex relationships between the nodes. The architecture consists of several steps: graph representation, message passing, node representation update, graph-level readout, and prediction or output. A data representation system is a GNN with an Adam optimizer, 100 epochs, a learning rate of 0.001, and entropy loss.

Results

The network has 108 nodes, 134 edges, and 2.444 neighbors, with a diameter of 4, radius of 2, and characteristic path length of 2.635. It lacks clustering, sparse connectivity, wide connection variation, and moderate centralization. The GNN model's drug-gene interactions demonstrate high precision, recall, F1 score, and accuracy, with a high sensitivity to true-positives and low false-negatives.

Conclusion

The study employs a GNN model to predict drug-gene interactions in the Wnt/β-catenin pathway, demonstrating high precision and accuracy, but further research is needed.

## Introduction

The Wnt/β-catenin pathway is crucial in bone formation and remodeling. It plays a central role in osteoblast differentiation, bone remodeling, and skeletal homeostasis. The pathway initiates with the binding of Wnt ligands to specific Frizzled receptors and coreceptors, triggering a cascade of intracellular events that stabilize β-catenin [1]. The activation of this pathway leads to an increased expression of key osteoblast-specific markers, while its inhibition inhibits osteoblastogenesis. The Wnt-β catenin pathway also regulates bone remodeling and homeostasis by influencing osteoblast and osteoclast activity. The dysregulation of the Wnt/β-catenin pathway has been associated with bone-related diseases, such as osteoporosis, osteoarthritis, and osteosarcoma. Several strategies have been explored to modulate Wnt-β catenin signaling, including Wnt agonists, inhibitors of Wnt antagonists, and small molecules targeting key components of the pathway.

One previous study showed that LAMP2A overexpression [2,3] in human breast cancer stem cells (mBMSCs) promotes osteogenesis by upregulating active-β-catenin levels and inhibiting tumor necrosis alpha (TNF-α)-induced inhibition, protecting bone healing from inflammation and mitigating osteoclast hyperactivity. In type 2 diabetes mellitus (T2DM) mice, inhibiting the Wnt3a/β-catenin pathway recently reduced bone formation, leading to osteoporosis. Downhill running activates the pathway, improving bone structure and reducing body weight. These studies demonstrate the importance of Wnt-β signaling in bone formation. Polydatin [4] promotes the proliferation and osteogenic differentiation of hBMSCs through the BMP2-Wnt/β-catenin signaling pathway. It enhances proliferation and alkaline phosphatase (ALP) activity, upregulates osteogenic gene expression, and is blocked by Noggin and Dickkopf-1 (DKK1), inhibitors of the BMP pathway and Wnt/β-catenin pathway. EGF-Like Domain Multiple 6 (EGFL6) enhances angiogenesis and osteogenic differentiation through Wnt/β-catenin signaling, enhancing bone formation and potentially treating fracture healing and bone defect restoration by increasing EGFL6 secretion. These studies pointed to the drugs and genes involved in Wnt/β-catenin-based bone formation.

The drug-gene interactome [5,6] is a complex network of interactions between drugs and genes that underlie the pharmacological effects of drugs [7,8]. It encompasses drug metabolism, targets, and drug transporters, essential for understanding drug action, response, and potential adverse drug reactions. Genetic variations in Cytochrome P450 (CYP) genes can significantly impact an individual's ability to metabolize drugs, affecting drug efficacy and toxicity. Drug transporters, encoded by specific genes, control the movement of drugs across biological barriers, affecting drug absorption, distribution, and elimination. Despite progress, challenges remain, such as the complexity of gene-drug interactions, multiple genes, and environmental factors. Future directions include integrating large-scale genomic data, artificial intelligence, and machine learning techniques to identify novel drug-gene interactions and develop predictive models for drug response variability.

Deep learning can predict drug-target interactions, analyze gene expression data, and predict drug response variability based on an individual's genetic profile. It can also help understand drug toxicity mechanisms by analyzing relationships between drugs, genes, and adverse drug reactions. Graph neural networks (GNNs) [9] are a powerful tool for analyzing complex drug-gene interactions. They operate on graph-structured data, making them ideal for modeling drug interactions and target genes. GNNs can capture structural and relational information in a graph, enabling more accurate predictions and deeper insights [5]. They can incorporate a graph structure into the learning process, capturing complex dependencies and propagating information throughout the graph. GNNs have been successfully applied to predict drug-target interactions, analyze complex interactions between drugs, genes, and biological pathways, and model drug response variability based on an individual's genomic profile. However, challenges such as large-scale datasets and limited interpretability make understanding the underlying mechanisms of drug-gene interactions difficult. Despite these challenges, GNNs have shown great potential in drug-gene interactions, providing more accurate predictions, identifying novel drug-target pairs, and enabling personalized drug therapy. Few studies explore graph-based neural networks in drug-gene interactions in Wnt/β-catenin-related bone formation. So we aim to predict drug-gene interactions of Wnt/β-catenin pathway in bone formation through a GNN.

## Materials and methods

Data preparation

Drug-gene interactions of Wnt signaling were downloaded from https://www.probes-drugs.org/ [10]. The data set contains 135 drug-gene datasets that were annotated and preprocessed. Outliers were deleted, data was labeled, and modes of action (MOA) such as agonist, antagonist, inhibitor, blocker, and allosteric activity are among the drug-gene interaction types in this dataset.

Cytoscape

Cytoscape [11] is a powerful tool for building drug-gene interactions and studying their biological characteristics. Data was imported into the program, and nodes representing drugs and genes and edges representing their interactions were created. It offers plugins that measure network centrality, identifying the key players or hubs with significant regulatory roles. It also helps detect network modules or communities, groups of drugs, and genes with interconnected relationships. Cytoscape also generates visually appealing visualizations of the drug-gene interaction network, presenting the network structure, highlighting important nodes, and facilitating interpretation and communication of analysis results. This platform enables researchers to understand the intricate connections between drugs and genes in biological systems. A layout algorithm to visually organize the nodes and their interactions was used. The network's biological characteristics were used from Cytoscape plugins or tools, such as network centrality measures and network modules. Finally, visualizations of the drug-gene interaction network were generated and the results were analyzed.

Graph neural networks

Data Preparation

Graph data was prepared for node, genes, and drugs as node, target_type (type of interactions: single protein, protein complex, protein-protein interactions) as edge, mode of action as edge weight, with human, activity_biochemical, activity_cell as node features and the interaction type was set as a target.

GNN Architecture

GNNs are designed to operate on graph-structured data, capable of learning complex relationships between nodes. Their architecture consists of several steps: graph representation, message passing, node representation update, graph-level readout, and prediction or output. The first step involves representing the input graph as nodes and edges, each with a feature vector representing its attributes. The second step involves aggregating information from the neighboring nodes, updating their representation, and generating a graph-level representation. The final step is to apply a neural network layer or classifier to generate desired outputs, which was done using Python.

Hyperparameter tuning is a crucial step in optimizing the performance of GNNs, as these variables significantly influence the model's behavior and performance. Bayesian optimization is a widely used technique for hyperparameter tuning, utilizing prior evaluations to create a probabilistic model, which is particularly beneficial for expensive hyperparameter configurations like GNN training on large datasets. A data representation system is a GNN with an Adam optimizer, 100 epochs, a learning rate of 0.001, and entropy loss. It consists of a graph structure with nodes and edges, node features, message passing, graph convolution, readout/pooling, output layers, the Adam optimizer, epoch loss, learning rate, and entropy loss. The structure captures relationships between elements, while the loss function measures the discrepancy between predicted and actual values. The learning rate determines the step size for updating model parameters, while entropy loss measures the difference between predicted and true distributions.

Several libraries and frameworks with automated hyperparameter tuning for GNNs using algorithms like random search were used. Common hyperparameters, including learning rate, layers, hidden units per layer, dropout rate, weight decay, batch size, and activation functions, were applied. Hyperparameter tuning was performed separately or through cross-validation for unbiased evaluation. A few evaluation metrics were also used to verify the results. Accuracy measures the proportion of correctly classified nodes or graphs used for the classification tasks. This study used precision, recall, and F1-score for binary classification or imbalanced datasets. The receiver operating characteristic (ROC) curve represents the trade-off between the true- and false-positive rates for different classification thresholds generated. Area under the curve (AUC)-ROC summarizes a classifier's performance over all the possible classification thresholds used to compare models and analyze the results.

The data representation system consists of a graph structure with nodes and edges, node features, message passing, graph convolution, readout/pooling, output layers, the Adam optimizer, epoch loss, learning rate, and entropy loss. The structure captures relationships between elements, while the loss function measures the discrepancy between the predicted and actual values. The learning rate determines the step size for updating model parameters, while entropy loss measures the difference between the predicted and true distributions.

## Results

**Figure 1:**
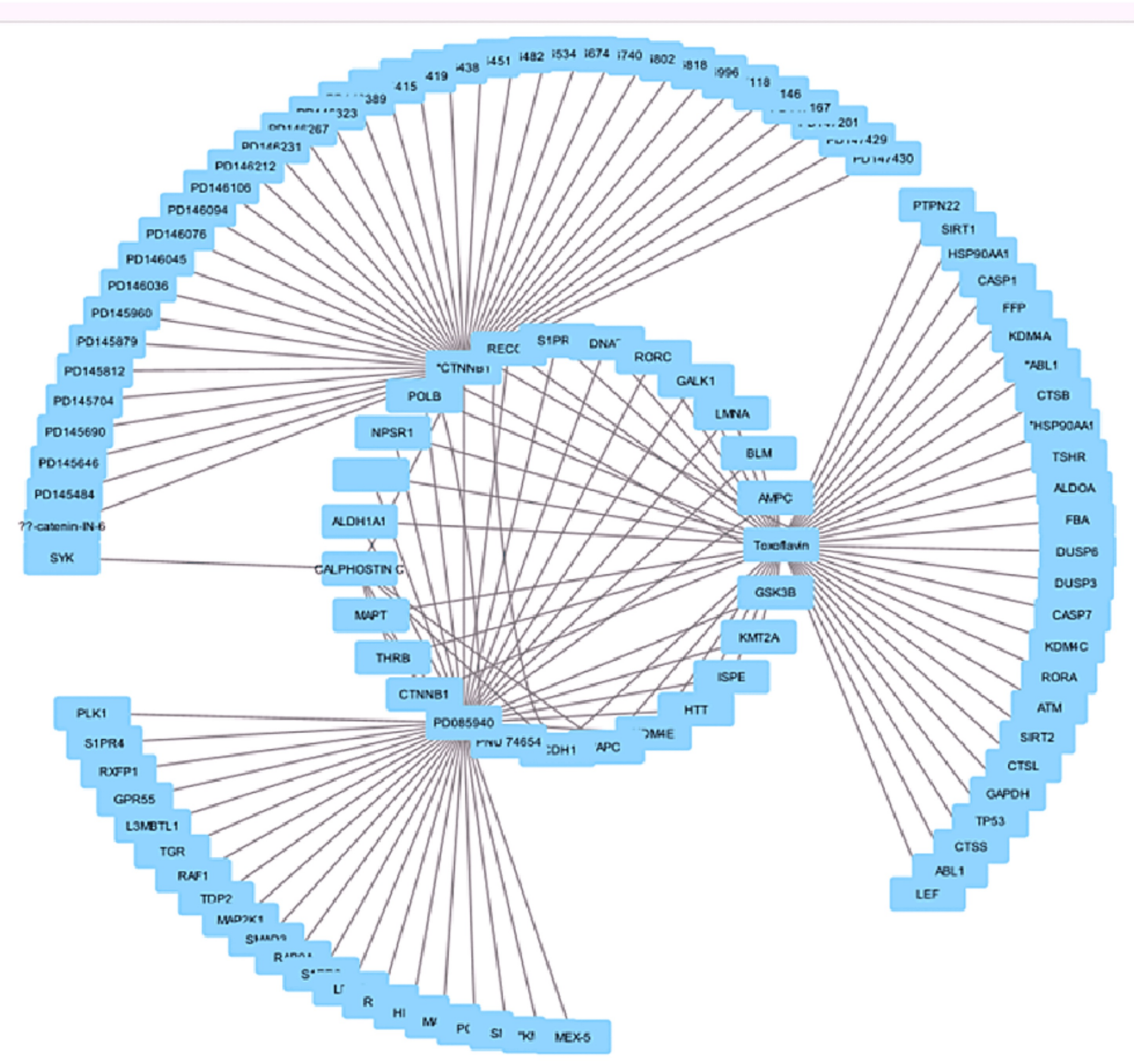
The drug-gene interactome of the Wnt/β-catenin pathway

The results show that the drug-gene interaction network includes the number of nodes, edges, average neighbors, network diameter, radius, characteristic path length, clustering coefficient, network density, network heterogeneity, network centralization, connected components, and analysis time. The drug-gene interactome of the Wnt/β-catenin pathway is demonstrated in Figure 1. 

The network has 108 nodes, 134 edges, and an average of 2.444 neighbors. The network has a network diameter of 4, a network radius of 2, and a characteristic path length of 2.635. The clustering coefficient indicates no clustering, and the network density indicates sparse connectivity. The network heterogeneity indicates wide variation in connections, while the centralization value indicates moderate centralization. The connected components indicate that all the nodes are connected. The analysis time is 0.144 seconds, indicating a moderate level of centralization. The GNN model's evaluation metrics show high precision, recall, F1 score, and accuracy. Precision measures true-positive instances, with 0.875 indicating 87.5% of class 0 instances are true-positives, indicating a low number of false-positives. Recall, or sensitivity, measures the accuracy of a model in identifying positive instances, with a recall of 0.875 indicating a low number of false-negatives. The F1 score, a harmonic measure of precision and recall, indicates a good balance between identifying true-positives and minimizing false-positives and -negatives. The model's accuracy, measured at 0.8888889 or 88.9%, accurately classifies 88.9% of instances across all classes as depicted in Table 1. 

**Table 1:** The accuracy of the graphic neural network (GNN) model Class: A category or label in a dataset. Precision refers to the accuracy of positive predictions; Recall is the ability to find all positive cases; F1 score shows the balance of precision and recall; and Accuracy means the overall correctness of predictions.

Class	Precision	Recall	F1 Score	Accuracy
0	0.875	0.875	0.875	0.8888889

**Figure 2:**
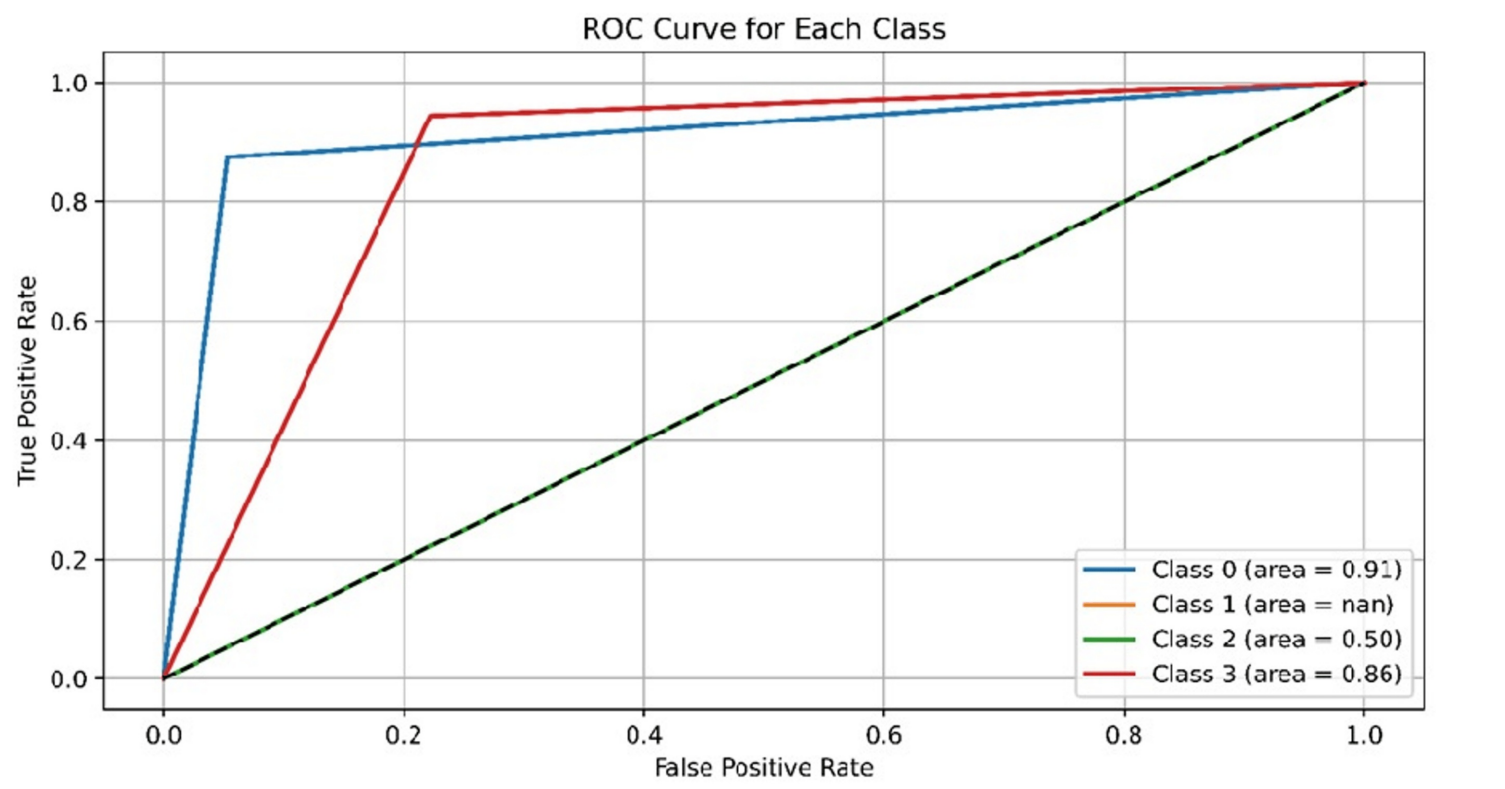
The ROC Curve for each class, representing the performance of a binary classifier system. The AUC values range from 0 to 0.91, with Class 3 performing well. AUC: Area under the curve; ROC: receiver operating characteristic

The ROC curve for each class was made, representing the performance of a binary classifier system. The AUC values range from 0 to 0.91, with Class 3 performing well, as depicted in Figure 2.

**Figure 3:**
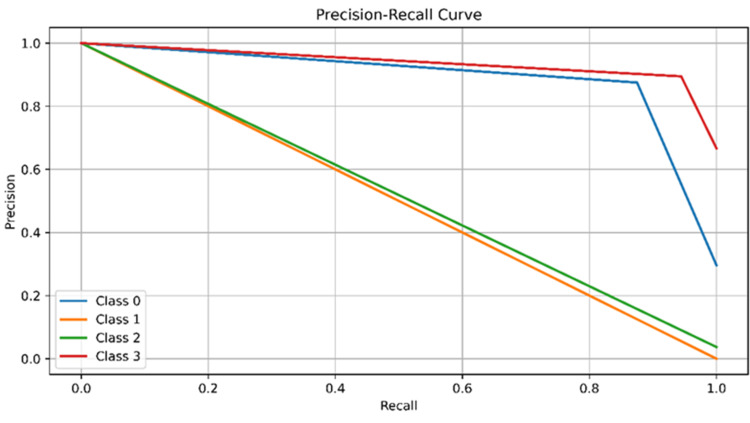
The Precision-Recall curve graph, which shows the performance of different classes (Class 0 to Class 3) based on precision and recall values. The x-axis represents recall, ranging from 0.0 to 1.0, and the y-axis represents precision, ranging from 0.0 to 1.0, in each class. Class 0 exhibits high precision and recall, while Class 1 maintains high precision. Classes 2 and 3 show decreased precision with recall, particularly in class distribution imbalances

The Precision-Recall curve graph, which shows the performance of different classes (Class 0 to Class 3) based on precision and recall values, was generated. The x-axis represents recall, ranging from 0.0 to 1.0, and the y-axis represents precision, ranging from 0.0 to 1.0, in each class. Class 0 exhibits high precision and recall, while Class 1 maintains high precision. Classes 2 and 3 show decreased precision with recall, particularly in class distribution imbalances, as depicted in Figure 3. 

**Figure 4:**
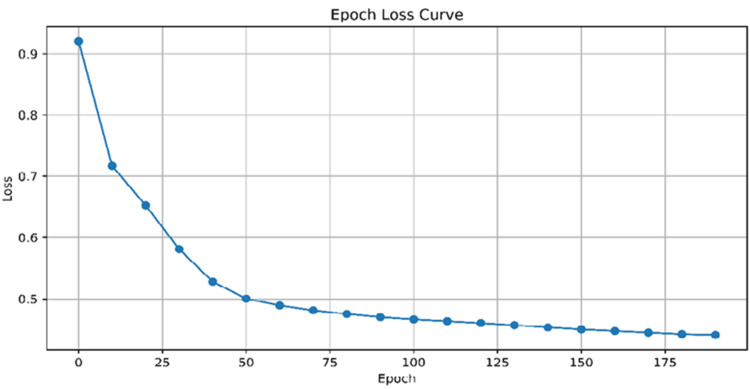
"Epoch Loss Curve" with loss values ranging from 0.5 to 0.9 and epoch values from 0 to 200. The graph shows a converging model learning from data as training progresses, minimizing loss function over time

An "Epoch Loss Curve" with loss values ranging from 0.5 to 0.9 and epoch values from 0 to 200 was generated. The graph shows a converging model learning from data as training progresses, minimizing loss function over time, as depicted in Figure 4.

## Discussion

The Wnt/β-catenin signaling pathway is crucial for bone formation and development, regulating osteoblast differentiation and proliferation. The key genes involved in this pathway include Wnt ligands, Frizzled receptors, LRP5 and LRP6, Disheveled proteins, Axin, and T-cell factor/lymphoid enhancer factor (TCF/LEF) transcription factors. Wnt ligands, such as Wnt1 and Wnt3a, initiate the pathway's activation, leading to the stabilization and nuclear translocation of β-catenin. Frizzled receptors, such as Frizzled-1, 2, and 9, regulate osteoblast differentiation and function. LRP5 and LRP6 are coreceptors for the Wnt/β-catenin pathway, and mutations in these genes can lead to altered bone mass and increased susceptibility to bone disorders. The top hub genes from this study include MAP2K1, LMNA, HSP90AA1, GAPDH [1,12,13] using Cytohubba, a Cytoscape extension, as shown in Figure 1.

MAP2K1 (MEK1) is a protein kinase that activates MAPK1, activating downstream effectors involved in bone formation. LMNA (LMNA) is involved in bone development and maintenance, interacting with β-catenin and modulating its activity. HSP90AA1 (HSP90AA1) stabilizes β-catenin, promoting its nuclear translocation and activation. SIRT1 (Sirtuin family) deacetylases regulate the Wnt/β-catenin pathway by deacetylating β-catenin, leading to its degradation and attenuation. TP53 (p53)(2) interacts with β-catenin and negatively regulates the Wnt/β-catenin pathway, impacting bone formation and homeostasis. Glyceraldehyde 3-phosphate dehydrogenase (GAPDH, a glycolytic enzyme) is a regulator of the Wnt/β-catenin pathway, promoting its nuclear translocation and activation of target genes involved in bone formation. TIEG1 [14] regulates bone Wnt signaling, affecting β-catenin nuclear localization and interacting with Lef1 and β-catenin. Inadequate TIEG1 expression may lead to osteopenic phenotype in TIEG1 KO mice and osteoporosis in humans, and a recent study found that osteoprotegerin (OPG) and RANKL expression are controlled by Wnt/β-catenin and BMP-2 signaling. Wnt/beta-catenin induces OPG expression, while BMP-2 enhances its activation. The study highlights two transcription pathways involving the OPG promoter.

Graph analysis is crucial for understanding drug-gene relationships, providing insights into drug mechanisms, targets, interactions, and genetic variations. It maps these associations as nodes and edges, identifying drug-gene pairs and their strength. Graph analysis can integrate multiple data sources, revealing hidden relationships between drugs and genes. This method helps researchers prioritize drug-gene pairs for further investigations. Functional Representation of Gene Signatures (FRoGS) is a deep learning approach that uses biological functions to represent gene signatures, enabling more effective compound-target predictions and integrating additional pharmacological data, proving useful in bioinformatics applications and revealing new relationships in large-scale omics studies [15,16]. One previous study proposes SGCLDGA [17], a novel computational model that uses GNN and contrastive learning to predict drug-gene associations similar to this study with an accuracy of 88%. Another study uses a deep variational autoencoder model to predict drug responses in cancer treatment outcomes. It compresses gene data into low-dimensional latent vectors, improving the accuracy similar to a good accuracy obtained in this work. The proposed approach uses an ensemble of generalized tensor decomposition (GTD) and multi-layer perceptron (MLP) to predict drug-gene-disease associations, enhancing flexibility and non-linearity [18]. Experimental results show a 7% improvement in triple association prediction and competitive accuracy in pairwise predictions [19] with similar, better accurate predictions for Wnt/β-catenin.

Drug-gene interactions of this GNN model exhibit high precision, recall, F1 score, and accuracy, with a high sensitivity to true-positives and low false-negatives. Its F1 score balances precision and recall, and its accuracy of 88.9% across all classes is impressive, as is the target type shown in Figures 2-4, and Table 1. Future GNN models should include dataset expansion, incorporating domain knowledge, using transfer learning techniques, and fine-tuning hyperparameters.

The current study demonstrates the potential of GNNs in predicting drug-gene interactions within the Wnt/β-catenin pathway, which is crucial for bone formation. Despite the high precision, recall, F1 score, and accuracy obtained in this study, its limitations must be acknowledged. The model's performance, while impressive on the training dataset, may not generalize well to unseen data due to potential overfitting and the inherent complexity of the Wnt/β-catenin signaling pathway. Moreover, the scalability of the model is challenged by the computational demands of processing large, intricate graphs, which may limit its application to more extensive datasets. Moreover, the interpretability of GNNs remains a significant hurdle, as their deep architecture often obscures the understanding of the underlying mechanisms driving predictions. Finally, potential biases in the data, such as imbalanced class distributions or incomplete interaction annotations, could influence the model's predictions, highlighting the need for further refinement and validation in diverse biological contexts.

## Conclusions

This study explores using a GNN model to predict drug-gene interactions in the Wnt/β-catenin pathway. The model's high precision, recall, F1 score, and accuracy suggest its ability to predict these interactions accurately. The Wnt/β-catenin pathway is crucial for various biological processes, including embryogenesis, tissue homeostasis, and bone development. Dysregulation of this pathway is linked to bone-related disorders like osteoporosis, periodontitis, and bone metastases. Understanding this pathway's complex interactions between drugs and genes is crucial for developing targeted therapies. The model's high precision, recall, F1 score, and accuracy show that it can uncover novel drug-gene interactions and potentially identify new therapeutic targets for bone-related disorders. However, further research is needed to validate and generalize these findings, replicate the analysis in other pathways, and experimentally validate the predicted interactions. The study of drug-gene interactions holds great promise in advancing our understanding of disease mechanisms and improving therapeutic interventions for the Wnt/β-catenin pathway.
